# Young adults’ experiences of living with paediatric acute-onset neuropsychiatric syndrome. An interview study

**DOI:** 10.1080/17482631.2023.2267268

**Published:** 2023-10-10

**Authors:** Ulla-Karin Schön

**Affiliations:** Department of Social Work, Stockholm University, Stockholm, Sweden

**Keywords:** PANS, experiential knowledge, collaborative care, mental healt care, epistemic injustice

## Abstract

**Aim:**

This article explores experiential knowledge of living with paediatric acute-onset neuropsychiatric syndrome (PANS), and the factors that are associated with perceived good care.

**Methods:**

Ten people with lived experience of PANS participated, five women and five men aged 19–34. Semi-structured interviews were used to explore their experience of living with PANS and their encounters with healthcare. Thematic analysis was carried out to identify central themes in the transcribed interviews.

**Results:**

The study revealed a group of young adults living fairly isolated lives, dependent on care from relatives. To them, the illness was a tangible presence. They perceived a lack of knowledge among healthcare staff on PANS in healthcare, and negative consequences linked to this. In addition, their experience-based knowledge of their own illness is devalued in healthcare encounters. A feeling of being pushed around in healthcare, without anyone taking responsibility for the treatment, emerged in the interviews. The participants emphasized the need for increased knowledge among staff to identify PANS and be able to offer effective treatment.

**Conclusion:**

There is a need to increase the knowledge about PANS in healthcare and to coordinating care between neurology, immunology and psychiatry. To be able to offer evidence-based care to children with PANS, in-depth knowledge is needed about aetiology, treatment effects, and user experiences and preferences.

## Background

This article discusses experiential knowledge of living with paediatric acute-onset neuropsychiatric syndrome (PANS). Paediatric acute-onset neuropsychiatric syndrome is an umbrella term for a range of symptoms that occur in varying degrees of severity. Experiences of an explosive onset of obsessive-compulsive symptoms and developments of tics are described, and both the affected persons life and their family’s lives are influenced in a substantial way (Dolce et al., 2022). The illness occurs suddenly and manifests itself through symptoms such as anxiety, compulsion, tics, and eating disorders. The syndrome mainly affects children, but some studies show that adults can also have PANS (Endres et al., 2022). On the other hand, some adults have PANS symptoms that they have had since childhood, and for which they have not received treatment. In some of these adults, PANS is diagnosed retrospectively and treatment can be offered.

In recent years, a series of studies and reviews have been carried out that summarize the state of knowledge regarding what can cause PANS and available assessments and treatments (see, e.g., Endres et al., 2022; Howes et al., 2021). Thus, in North America, guidelines were published in 2017 for the treatment of PANS based on research and clinical experience by the PANS Research Consortium (Cooperstock et al., 2017; Frankovich et al., 2018; Sigra et al., 2018; Thienemann et al., 2017). In Sweden, as in many other countries, there are currently no national guidelines and the treatment and care for PANS has been highlighted to be in urgent need of enhanced knowledge. However, local clinical guidelines based on the international research available, have been provided. Previous research recommends an individualized treatment plan according to the patient’s needs and progress, with simultaneous focus on symptom relief and treatment of the underlying causes. Antibiotics are recommended when there is a known or suspected bacterial infection. In addition, information, support, and psychotherapies (e.g., Cognitive Behavioral Therapy (CBT)) are recommended early in the course, to alleviate symptoms. Anti-inflammatory treatment is recommended for mild and moderate symptoms. For more severe symptoms, immunomodulating treatment is recommended, including intravenous immunoglobulin (IVIg). The need for close collaboration between several specialities, such as child psychiatry/neuropsychiatry, child neurology/medicine, and immunology/rheumatology, is emphasized.

Previous research on PANS mainly focused on prevalence, aetiology, and treatment effects. When it comes to subjective experience of the illness, the knowledge is limited. And more specifically, when it comes to what it is like to be a young adult with experience of PANS, it is an unexplored area. Some research has been conducted based on the effects of PANS on the family (Frankovich et al., 2018; Ringer & Roll-Pettersson, 2022). In these studies, significant negative consequences of PANS syndrome on the family have been illustrated. This is explained by the long-term nature of the condition, a perceived lack of control over symptoms, and major challenges in accessing medical treatment. Frankovich and colleagues (Frankovich et al., 2018) report that families with a child with PANS are affected significantly more than other families with children with chronic childhood illnesses regarding emotional wellbeing, which entails a higher level of suffering. The family’s functioning is affected by a lack of support from medical care, from the children’s school, and also from family and friends. Furthermore, research shows that the neuropsychiatric symptoms, which can be severe, can be a particularly challenging aspect of PANS for parents and siblings (Frankovich et al., 2018).

The difficulty in accessing adequate care and treatment is therefore described as one of the most difficult challenges of living with PANS in the family. This may be due to several reasons: For example, the syndrome is still considered new and unknown; the condition addresses a need that conflicts with the organization and specialization of care; also, it may be a “controversial syndrome” (Frankovich et al., 2018). Previous research on controversial syndromes such as chronic fatigue syndrome (CFS) or multiple chemical sensitivity (MCS) illustrates how people who suffer from these syndromes often experience their illness as being questioned or denied (Armentor, 2017; Finell et al., 2018). Their symptoms are judged by healthcare staff to be psychosomatic, which leads to them being offered psychiatric diagnoses without a neurological examination being considered (Finell et al., 2018; Kornelsen et al., 2016). Moreover, their motives are questioned, their needs remain unknown, and their right to care is denied (Finell et al., 2018; Montali et al., 2011). These experiences can be considered a kind of epistemic injustice (Fricker, 2007) where people’s expressed suffering is questioned or dismissed by the healthcare system, which has the power to either recognize or discredit this suffering (Blease et al., 2017; Buchman et al., 2017; Fricker, 2007).

There are of course differences between CFS, MCS, and PANS regarding severity, symptoms, and everyday function. Yet the treatment in care and the experience of having their knowledge and care needs rejected may be similar. This article explores lived experience of PANS, and investigates which factors are associated with perceived good care. Experiential knowledge is limited in previous research on PANS, but is valuable for deepening the knowledge about how care and support can be developed to provide suitable care for children and adults with PANS.

## Method

Qualitative, semi-structured individual interviews on personal experience of living with PANS were conducted to capture experience and knowledge on a range of topics such as everyday life and encounters with healthcare. The participants were recruited through an advertisement in a newsletter from a user organization for people with experience of PANS. The advertisement sought adults with experience of living with PANS who were willing to participate in an interview study. Those interested were asked to contact the researcher via email or phone for more information. Six months later, two further reminders were sent with the intent of reaching a few more persons willing to participate. A total of ten people with lived experience of PANS finally participated, five women and five men aged 19–34. According to the participants own stories, all had experience of having had PANS-like symptoms already in childhood or early adolescence. The interviews took place during February-September 2021, and were conducted at a location chosen by the participant. This resulted in most interviews being conducted digitally via Zoom. Two interviews were conducted as telephone interviews and two took place person to person. The interviews lasted between 50 and 80 minutes and were recorded and later transcribed verbatim. The interviews were de-identified during transcription and each interview was given a number.

### Analysis

A thematic analysis was then carried out based on Braun and Clarke (2006). Their analysis model consists of five steps, where step one is about getting to know the material that made up the recorded interviews, by transcribing it verbatim and reading it in its entirety several times. The second step involves developing initial codes in a systematic way throughout the material; this was done using the programme NVIVO (QSR International, Burlington, MA, USA). In the third step, the codes were further analysed in NVIVO and grouped into possible subthemes. In step four, the themes were reviewed and revised to some extent by comparing them with each other and checking them against the printed interview text. In the final, fifth, step, themes were defined and named, and the main features were described in detail. These themes form the basis for the findings below.

### Ethical considerations

This study was approved by the Swedish Ethical Review Authority (case number: 2021–06902–02). Participation in the research was confidential and voluntary. The participants were given a detailed explanation of the study and its aim. They were also informed of the data collection procedures. Individuals who agreed to participate were requested to complete a written informed consent form. The participants were informed that they could withdraw from the study whenever they wished to do so. In connection with the interview, the participants were again informed that their participation was anonymous and that names and place names that would risk identifying them would be changed or removed in connection with the transcript. In addition, they were informed that participation was completely voluntary and that there was the possibility to skip questions or cancel or break off the interview at any time. All participants were offered the opportunity to read their interview transcript. Six participants read their transcripts but had nothing further to add or withdraw.

## Results

Two overarching themes emerged from the analysis. The first, *Living outside the expected life*, concerns the participants’ descriptions of how they lived outside the life they had expected to live (and the life that others did live) as adults, with vulnerable and unpredictable health conditions, but also with inability to conform to expectations of care in terms of diagnostic criteria and healthcare staff expertise. The second theme, *Co-production of knowledge-based care*, features the participants’ descriptions of the need for unconditional care from several care specialities and the value of the lived experience.

### Living outside the expected life

A majority of the participants had been diagnosed with PANS as children (i.e., before age 18); others were diagnosed as adults. Regardless of when they received their diagnosis, they had lived with a severe illness which they called a “PANS-like illness” for many years, before PANS was recognized by healthcare staff. The participants described a life situation still characterized by the illness. Five lived at home with their parents, four had their own accommodation, and one was cohabiting with a partner. All received a lot of support in their everyday life. The support came from healthcare contacts or other authorities, but they also received support in daily care and in the form of meaningful companionship. Participation in work, employment, or studies was low among the participants, with only one of them studying, though with an adapted course plan, and one working part-time. The participants described how they spent their time at home, mostly on their own. They talked about their lives as apart from the life that many of their peers lived:

Though it has been 15 years since I got sick, my life has become more and more confined. You could say it is deprived in some way. I do very little. I very rarely get out of the door and I have no activities that I do. (IP2)

The narratives described an existence where the condition controlled the content of each day and also the ability to perform activities. The illness was described as coming in fits, starting suddenly, and making the participants unable to cope with daily routines. They struggled daily with the consequences of PANS; at the same time, they described periods in which they had the ability to do more than in the past. Activities such as going to a café, or spending time with others with whom they shared music or other common interests, were highlighted as examples of improvement. These activities would not have been possible previously, when they had felt even worse.

Right now, my plan is to only go out to the stables one day a week, for a certain time. Because that’s as much as I can handle, right now. Before, I couldn’t do anything at all basically … . I’m out shopping sometimes and occasionally I meet friends, too, but I didn’t do that at all before. (IP4)

But it is not only the expected life of an adult that the participants had experience of “standing outside of”. They also described that their suffering did not conform to explanatory frameworks of healthcare. Years of intense seeking for care and information in somatic and psychiatric care had finally led to an understanding of the illness they suffered from, and of their behaviours and sudden inabilities. During this period, they had received several different psychiatric diagnoses, all characterized by symptoms that did not fully comply with their own, and received medical treatment that they did not perceive to provide any appreciable relief. The participants described experiences of “despair” and “being thrown around” within the system of care. Their condition was understood as a “new illness”, which, according to the participants, meant that the healthcare staffs’ knowledge was limited. They saw this as the reason why PANS had not been considered in any of their numerous health encounters when they were children, despite the fact that the experience of such problems went back to as early as 9–10 years of age in some. Coming for an appointment and seeing a doctor who had not previously heard of PANS was an experience shared by all respondents:

Many doctors are not familiar with PANS. I don’t know if that’s because it hasn’t been an established diagnosis for long. Or possibly, that I saw the wrong doctors? (IP1)

Moreover, PANS was also described as a “controversial condition” that some doctors were described to question. Today, PANS is a so-called “diagnosis of exclusion,” which means that the diagnosis is reached by a process of elimination, when the problems experienced cannot be explained by any other condition or diagnosis. The participants talked about how they felt that there was an “emphasis on psychiatry” in the eyes of the healthcare workers, where established diagnoses such as autism and obsessive-compulsive disorder (OCD) were quickly put forward. The therapists’ frequency of working with these diagnoses also meant that the diagnoses were “close at hand” when the participants sought help:

But doctors you see at the health center have difficulty understanding this thing about obsessive and compulsive actions. And [they don’t understand] PANS at all. I should have come to a pediatrician’s office straight away when I was 14, but I was sent to child psychiatry instead and was given antidepressants and such. (IP8)

At the same time, there was an understanding among the participants that PANS “can be easy to misdiagnose,” as many expressions of PANS are described as psychiatric, for example severe anxiety, tics and compulsion. On the other hand, the participants questioned why therapists stuck with a certain treatment over time, if it had no effect.

### Co-production of knowledge-based care

What the participants wished for, and in many cases were still searching for, was knowledge-based care where healthcare staff seek collaboration with colleagues in other medical specialities and with the patient and her or his relatives.

Some of the participants had previous experience of temporary treatment with antibiotics in Norway or Denmark, which they felt had had positive effects on their wellbeing. These treatments had given them the experience of meeting a “knowledgeable doctor” or an “expert on PANS,” which had been a relief both in the sense that they “felt believed” for once in their description of the syndrome, and also in the sense that they had access to an “effective treatment.” A few of the participants had been part of Swedish research projects where they had been offered medical treatment with rituximab, which had made them feel much better. But the projects had been time-limited and continued treatment had proved difficult to obtain, despite a clear sense of improvement. Therefore, for care to be perceived as “good,” the participants had to experience that the treatment offered was not “temporary” or time-limited. Those few who had experienced this described the relief of not having to emphasize their needs to healthcare staff and not having to “fight for treatment.”

So, this emotional turning point, that now I’m safe. It was in psychiatry [where I got this] treatment [IVIg]. And all the doctors approved it, it’s just anchored. And I have my doctor fighting for me. She does what is best for me and I know she is there [for me]. (IP3)

In addition to unconditional care, the need for a broad perspective on the syndrome was emphasized. In their narratives, the participants made a distinction between treatment “for PANS symptoms” and treatment “to function in everyday life.” The treatment described as effective involved immunomodulating treatments with intravenous immunoglobulin (IVIg) with the antibody medicine rituximab, or with cortisone. These treatments were said to have had a great effect:

“It was a treatment over several days. Then the third day it got silent in my head, it was silent. It was a big struggle to get treatment. … but it’s like night and day” (IP3).

The participants described the effects of certain treatment as “absolutely decisive.” They had developed expertise on how it worked for their body, and had adjusted their social life accordingly. In addition to these treatments, examples of participants taking control of their own treatment was illustrated in having their tonsils removed or taking painkillers containing ibuprofen daily, as an anti-inflammatory treatment, as they had read that it had helped others with PANS to reduce the risk of relapse. Regarding treatment for dealing with everyday life, they referred to both CBT to develop strategies to deal with, for example, compulsion and anxiety, and more long-term forms of therapy. Those who had been offered longer-term therapy described it as having been a support in understanding previous illness experiences and struggles in managing the syndrome.

I think that the psychologist I had, went back a very long time in my illness and really tried to understand how it has been for me. It helped me a lot mentally./…/ I would say it hasn’t helped with the symptoms, but it has helped with something else. (IP9)

In addition to unconditional care and a desire for the healthcare staff to acquire a broad knowledge of their condition, the importance of a patient being respected as a person with lived experience was also highlighted. The participants had extensive experience of being ignored despite their extensive lived experience on a rare illness. They expressed that in healthcare encounters, it was made clear where the “expertise” lay, and that healthcare staff clearly articulated that “we know more than you know.”

The experience that assigned diagnoses “did not seem right” or “did not fit at all” made relatives, in this study mainly mothers, carry out their own research for information on the Internet, through support groups and in scientific search databases. However, suggesting possible diagnoses and treatment methods to doctors and other healthcare workers was not necessarily welcomed by the healthcare workers, who often dismissed relatives and the participants themselves despite their experience and knowledge. Several of the participants expressed that their mothers had been badly treated by the healthcare system when they had questioned diagnoses and expressed mistrust regarding treatment, above all in situations when they had tried to bring forward international and research-based knowledge about PANS and possible treatments.

My mother has done a lot. She is the one who has had to manage almost all contact with the social insurance fund, doctors, calling about appointments, and so on. Searching around without getting help … . It’s thanks to her that I am better. She has helped me get antibiotics from this doctor in Norway, because no one wanted to prescribe this in Sweden. When I was at my worst, she was like a carer. (IP4)

Several of the participants expressed explicitly that it was thanks to their mothers’ “tireless struggle” that they were alive today.

## Discussion and concluding remarks

This article explores experiential knowledge on what it is like to live with PANS as an adult, and which factors are associated with perceived good care. The results reveal that the studied group of adults were living fairly isolated lives and were dependent on care from their relatives. The illness still had a tangible presence. Factors such as an increased knowledge of PANS among healthcare staff, access to effective treatment and a structure for receiving experience-based knowledge in healthcare are highlighted as central in the interviews.

A limitation in this study is the unilateral recruitment through a user organization. Recruiting via a user organization has advantages in terms of reaching an often hard-to-reach group. At the same time, it implicitly means that the participants themselves and/or their relatives are committed to the issue and that that commitment is relevant right now. They have sought out support and may be representing a group more active in seeking support, or a group impacted most severely by PANS. There may be a risk that people who have recovered from PANS and have terminated their membership may not be reached. Likewise, the recruitment method excludes young adults with PANS who are unaware of the organization. The participants represent white middle class, with resourceful parents, which in this study excludes experiences of living with PANS in a vulnerable family.

Nevertheless, the results from the ten rich narratives about what it is like to live with PANS illustrate how the participants had important knowledge about factors contributing to improved health. They described what, based on their needs, characterizes good care. However, at the time of the interviews, only three of the participants had ongoing treatment which, they felt, targeted the PANS syndrome and had a noticeable effect. Others described a need for care that had not been met. However, most of the participants had at some point during their illness received care that had clearly improved their wellbeing, within the framework of research or in meetings with specific healthcare workers.

The participants emphasized a need for increased knowledge in healthcare staff to diagnose PANS and offer effective treatment. According to the participants, such treatment needs to span several specialist areas in healthcare, such as immunology, neurology, and psychiatry, to address the complex and individual needs of a PANS sufferer.

The described experiences confirm and deepens what has emerged from previous research on PANS interventions and healthcare needs (Cooperstock et al., 2017; Sigra et al., 2018; Thienemann et al., 2017), stating that, in order to be effective, the treatment of PANS needs to focus both on symptom relief and treatment of the underlying causes, as well as provide information, support, and psychotherapy early in the course of the syndrome (Endres et al., 2022; Howes et al., 2021).

This study describes a lack of knowledge on PANS in healthcare and illustrates the negative consequences of this lack. Children and adults with PANS are often denied a medical examination and care. In addition, their experience-based knowledge is devalued in the encounter with care.

One finding in this study was the participants’ feeling of being pushed around in care without anyone taking responsibility for their treatment. Experience of mistreatment or poor treatment among both the participants and their mothers was related in all the narratives and was described to lead to poorer health and a feeling of abandonment. Despite the fact that the participants had lived and managed their illness for many years, they perceived that their experience-based knowledge was not valued or even asked for by healthcare. On the contrary, the participants felt that their experience was diminished or dismissed. Likewise, the mothers of the participants had been dismissed when they had brought forward knowledge they themselves have found out about PANS and its treatment. The narratives thus depict a tangible knowledge injustice (Fricker, 2007). These findings yield the need for more research exploring the interplay of lack of evidence, organizational readiness and healthcare staff attitudes and how it effects the patients and their carers.

To be able to seek care and express own needs, it is important to have representation. Here, the participants’ mothers appeared as indispensable, both as representatives and as ambassadors of new knowledge. However, this representation seems to entail great difficulties in living a life including work and in finding time for socializing and other interests. This picture is also in line with previous research that specifically focused on the consequences of PANS on families (Frankovich et al., 2018; Ringer & Roll-Pettersson, 2022). These results raise the question of how things are going for people with PANS syndrome who do not have a parent with the resources or ability to shoulder a representative role and at times offer care around the clock.

To conclude, this article contributes new knowledge, from an experienced based perspective, how care and support can be developed to provide suitable care for children and adults with PANS.

## Biographical note

My research interests lie in the field of disability, especially disabled people’s opportunities to participate in social life, in meeting with social services and health care, as well as in education. In recent years, I have led interdisciplinary research projects focused on user-involvement, person-centred social practices, how digital support for clients/patients can promote participation, and how implementation of new interventions can be promoted in social services and health care. I also run a project that studies how young people without work and studies can be supported to make a move towards work, participation and a sense of belonging.
